# THE ROLE OF AREA AGENCIES ON AGING IN PUBLIC HEALTH VACCINATION CAMPAIGNS: FINDINGS FROM A NATIONAL SURVEY

**DOI:** 10.1093/geroni/igad104.0840

**Published:** 2023-12-21

**Authors:** Traci Wilson

**Affiliations:** USAging, Washington, District of Columbia, United States

## Abstract

When COVID-19 vaccinations became available, AAAs played a critical role in ensuring that older adults could receive the lifesaving vaccine. AAAs built on their existing service expertise and vast partnership networks to quickly implement vaccine outreach and education, scheduling support, transportation and other supportive services to attend the appointment, and age-friendly and culturally appropriate vaccination clinics. The CDC recognized AAAs’ early contributions and awarded $50 million to support continuation of these activities. In early 2023, the Aging and Disability Networks received an additional $75 million from ACL, awarded to USAging’s Aging and Disability Vaccination Collaborative, to fund local efforts across the United States to increase vaccine uptake among older adults and people with disabilities, targeting individuals from rural, minority, and disadvantaged backgrounds. This paper describes how AAAs were uniquely positioned to assume this expanded public health role and specific characteristics that contributed to their readiness. Data from the 2022 National Survey of Area Agencies on Aging (n=457 AAAs, 74% response rate) describe new and expanded AAA activities and partnerships that support public health efforts. We will discuss implications for community partnerships to improve public health and look ahead to measuring the impact of these vaccine promotion activities.

